# Bowel Perforation Secondary to Ponatinib Treatment in a Chronic Myelogenous Leukemia Patient: A Case Report

**DOI:** 10.7759/cureus.19949

**Published:** 2021-11-27

**Authors:** Asma Sallam, Giamal Edin Gmati, Refaat Salman

**Affiliations:** 1 College of Medicine, King Saud Bin Abdulaziz University for Health Sciences, Riyadh, SAU; 2 Division of Adult Hematology and Hematopoietic Stem Cell Transplantation, Department of Oncology, King Abdulaziz Medical City, Riyadh, SAU; 3 Department of Interventional Radiology, King Abdulaziz Medical City, Riyadh, SAU

**Keywords:** bowel perforation, tkis, blast crisis, cml, ponatinib

## Abstract

Chronic myeloid leukemia (CML) is a myeloproliferative neoplasm of hematopoietic cell origin.It arises from the translocation of chromosomes 9 and 22, with resultant Philadelphia (Ph^+^) chromosome that contains the BCR-ABL1 gene.^ ^CML has three phases: the chronic phase, the accelerated phase, and blast crisis. Tyrosine kinase inhibitors are used as the targeted therapy of CML. This report is about a 30-year-old male who is normally fit and well with no past medical history of note. He was diagnosed previously with CML and presented in a blast crisis. With this blast crisis at presentation, the patient was started on ponatinib. After 12 days from starting ponatinib, the patient presented with abdominal pain and vomiting. Imaging showed small bowel perforation, which required immediate surgery. The patient’s cardiovascular risk for such event was low and ponatinib was thought to be the most likely cause of this complication; thus, higher-risk patients for such ischemic events should be observed closely.

## Introduction

Chronic myeloid leukemia (CML) is a myeloproliferative neoplasm of hematopoietic cell line. It occurs when there is a translocation of the chromosomes 9 and 22, leading to acquiring Philadelphia (Ph+) chromosome that carries the BCR-ABL1 gene [1]. CML has three phases: the chronic phase, the accelerated phase, and the blast crisis [1,2]. Previously, CML was treated using hydroxyurea (HU), busulfan, interferon-based regimens, or hematopoietic stem cell transplantation (HSCT) [3]. The discovery of the BCR-ABL1 gene has led to the development of targeted treatment for CML [1]. Tyrosine kinase inhibitors (TKIs) are the mainstay medications used in the treatment of CML. Several TKIs have been approved for CML, and they are grouped into three generations. The first-generation medication is imatinib, which is the first-line medication for management of CML. The second-generation medications include dasatinib, nilotinib, and bosutinib, while the third-generation medication is ponatinib. For the initial management of CML, first- and second-generation medications were approved for use as first line, whereas in resistant cases, bosutinib and the third-generation medication ponatinib are reserved [4]. Each group of TKIs works differently on various binding sites of the tyrosine kinase enzymes, but the final effect is the same. Many side effects of TKIs are similar and are usually dose dependent; however, with the differences in their mechanism of action, each TKI has a variety of unique adverse effects [5]. For example, the epidermal growth factor receptor type of TKIs have more cutaneous side effects, while the vascular endothelial growth factor receptors are more likely to be associated with cardiovascular events, especially, hypertension [6,7]. Common side effects to all TKIs include fatigue, weight changes, fever, gastrointestinal symptoms, and others [7]. This case report will provide an insight into an adverse effect of ponatinib in a CML patient during his illness.

## Case presentation

**Figure 1:**
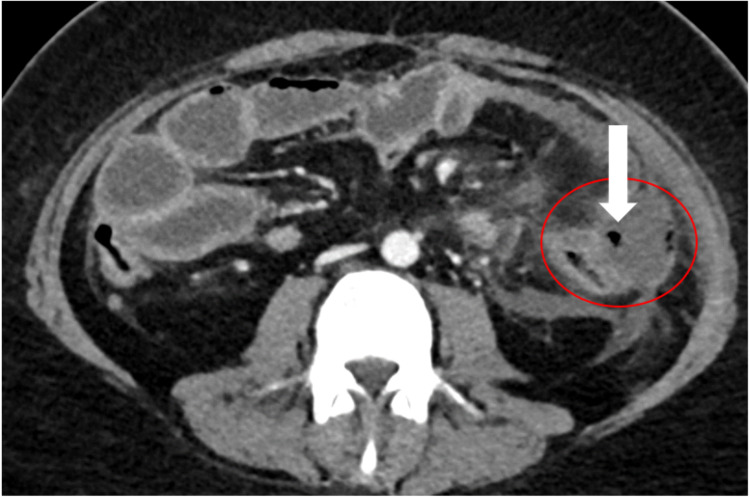
CT Image Axial image of CT scan in late arterial and early venous phase showing a 4x5 cm collection adjacent to the mid descending colon and proximal jejunal loops with multiple small air bubbles representing a bowel perforation and an infected collection (arrow). CT, computed tomography.

A 30-year-old male who is normally fit and well with no significant past medical history of note was diagnosed with CML in a different institution in 2015 and failed imatinib. Dasatinib was started after, but the patient was not compliant with the medication due to generalized bone pain and fatigability; however, due to the unavailability of detailed medical notes during this period we could not certify the clinical course of his disease at that time. He presented to our center in February 2020 with fever, productive cough, and chest pain. BCR/ABL P210 mRNA transcript was detected and estimated to represent 61% on international scale and complete blood count showed a hemoglobin of 90 g/L (135-180 g/L), platelets of 64×10^9^/L (150-400x10^9^/L), mean corpuscular volume of 97.5 fL (76.0-96.0 fL), and leukocytes of 54.50×10^9^/L (4.0-11.0×10^9^/L), with neutrophils 22.89×10^9^/L, lymphocytes 11.44×10^9^/L, blasts 27%, monocytes 1.09×10^9^/L, metamyelocytes 2%, myelocytes 3%, and bands 1.09×10^9^/L. This result indicates transformation to a blast crisis. The patient required intensive care unit (ICU) admission for pneumonia. HU 2000 mg was started as a cytoreductive agent followed by induction with idarubicin/cytarabine (7+3) protocol. Following remission status, which was confirmed by bone marrow examination, the patient was discharged on dasatinib with a plan to work him up for HSCT. However, two weeks later he presented with shortness of breath and chest pain. Chest X-ray revealed a worsening of bilateral pleural effusion and dasatinib was discontinued. TKI treatment was switched to ponatinib 15 mg then 30 mg plus aspirin, and the patient was discharged to be reviewed in the outpatient clinic. After 12 days from starting ponatinib the patient presented with abdominal pain and vomiting. Labs showed a neutrophil count of at least of 1.5x10^9^/L^ ^at presentation and for the previous four weeks and lactic acid of 5.8 mmol/L (0.50-2.20 mmol/L) which was normal until two days before presentation. CT abdomen showed scattered air foci indicating perforated viscus (Figure 1), and a diagnostic laparoscopy confirmed jejunal perforation around 20 cm from the duodenojejunal junction which was treated with small bowel resection. The histopathology revealed an active inflammation with submucosal edema and serositis, and neither thrombosis nor malignancy was identified. Since our patient had no other risk factors for bowel perforation, ponatinib had to be discontinued due to this complication and TKI treatment switched to nilotinib but again this had to be stopped due to prolonged Q-T interval. The patient’s general condition did not improve, and he needed a prolonged post-operative care in the ICU. Meanwhile his acute leukemia worsened with circulating blasts reaching 50% associated with multi-organ failure. Therefore, the patient was deemed not fit for further intensive therapy and his care was mainly palliative and he passed away in June 2020.

## Discussion

CML is a myeloproliferative neoplasm characterized by a reciprocal translocation of the chromosomes 9 and 22 creating a hybrid gene between BCR and ABL [8]. There are three phases of CML disease course. The first phase is the chronic one, which is described by the elevated number of white blood cells and proliferation of myeloid cells. This is the phase where most CML patients are categorized. The second phase is the accelerated phase, which occurs if the disease is not treated or is resistant to treatment. According to the World Health Organization (WHO) criteria, the accelerated phase includes 10-19% of blasts circulating or in the bone marrow, blood basophils of ≥20%, constant thrombocytopenia (<100×10^9^/L) not related to therapy, clonal chromosome abnormalities in Ph+ cells (CCA/Ph+) on treatment, thrombocytosis (>1000×10^9^/L) unresponsive to therapy, enlarged spleen, and elevated white blood cell count unresponsive to therapy [9,10]. The third phase is the blast crisis, which is defined by the presence of ≥20% of blasts in the peripheral blood or bone marrow. Additionally, the WHO adds to the definition extramedullary blast proliferation apart from spleen, and large clusters of blasts in the biopsy of the bone marrow. The treatment of CML requires the administration of TKIs, which are used in all phases of CML [9,10]. The progression to the blast crisis was inevitable prior to the introduction of TKIs [9,10]. Ponatinib, a third-generation TKI, is used in the treatment of the three phases of CML, refractory cases to first- and second-generation TKIs due to mutations, most famously T315I mutation, and Ph+ acute lymphoblastic leukemia patients [4,11]. The medication is designed to inhibit the function of native and mutated BCR-ABL tyrosine kinase [11,12]. Many side or adverse effects are known to be associated with the use of ponatinib, and they can be divided into cardiovascular adverse events (CAEs) and non-CAEs [13]. Some of the CAEs include arrhythmia, hypertension, left ventricular dysfunction, myocardial infarction, peripheral arterial disease, arterial thrombosis, and cerebrovascular accident, while non-CAEs include thrombocytopenia in 39.7%, anemia in 28.2%, abdominal pain in 33.3%, elevated lipase in 28.2%, and rash in 26.9% of patients [13]. The specific warning in the drug information in the Food and Drug Administration (FDA) label includes hepatotoxicity, arterial occlusive events, venous thromboembolic events, and heart failure. Multiple factors are known to increase the risk of developing these side effects such as dose, age, and previously diagnosed ischemic disease. For higher doses, the possibility of developing rash, pancreatitis, and heart failure increases. With any of these risk factors, care must be taken as the possibility of arterial occlusive events increases [13-15]. Bowel perforation as an adverse event of ponatinib is not widely reported; however, this specific complication can be found on the medication’s label and can occur due to the arterial occlusive events [14]. 

## Conclusions

In this case the patient developed jejunal perforation mainly due to bowel ischemia after 12 days of initiating the treatment. The patient was on prophylactic aspirin to prevent arterial occlusive events, and he had no history of venous thromboembolic disease in the past, yet he developed this adverse event. We suggest that patients with other risk factors and comorbidities should be screened for cardiovascular risk and followed up closely with regular history and serial physical examinations. Complaints made by patients on ponatinib should be taken seriously and appropriate tests should be considered according to the complaint. The benefits and risks of the medication and its dose adjustments should be taken into consideration. It might be wise to inform all patients about these side effects and how they could possibly present and to encourage them to visit their doctors whenever they experience new symptoms. Additionally, antiplatelet prophylaxis should be considered in every individual case, and with any previous thromboembolic phenomena anticoagulation needs to be added.

## References

[1] Chereda B, Melo JV (2015). Natural course and biology of CML. Ann Hematol.

[2] Kantarjian HM, Keating MJ, Talpaz M (1987). Chronic myelogenous leukemia in blast crisis. Analysis of 242 patients. Am J Med.

[3] Silver RT, Woolf SH, Hehlmann R (1999). An evidence-based analysis of the effect of busulfan, hydroxyurea, interferon, and allogeneic bone marrow transplantation in treating the chronic phase of chronic myeloid leukemia: developed for the American Society of Hematology. Blood.

[4] Jain P, Kantarjian H, Cortes J (2013). Chronic myeloid leukemia: overview of new agents and comparative analysis. Curr Treat Options Oncol.

[5] Roskoski R Jr (2020). Properties of FDA-approved small molecule protein kinase inhibitors: a 2020 update. Pharmacol Res.

[6] Ng CY, Chen CB, Wu MY (2018). Anticancer drugs induced severe adverse cutaneous drug reactions: an updated review on the risks associated with anticancer targeted therapy or immunotherapies. J Immunol Res.

[7] Kamba T, McDonald DM (2007). Mechanisms of adverse effects of anti-VEGF therapy for cancer. Br J Cancer.

[8] Adnan Awad S, Kankainen M, Ojala T (2020). Mutation accumulation in cancer genes relates to nonoptimal outcome in chronic myeloid leukemia. Blood Adv.

[9] Aladağ E, Haznedaroğlu İC (2019). Current perspectives for the treatment of chronic myeloid leukemia. Turk J Med Sci.

[10] Hochhaus A, Saussele S, Rosti G (2017). Chronic myeloid leukaemia: ESMO Clinical Practice Guidelines for diagnosis, treatment and follow-up. Ann Oncol.

[11] Hoy SM (2014). Ponatinib: a review of its use in adults with chronic myeloid leukaemia or Philadelphia chromosome-positive acute lymphoblastic leukaemia. Drugs.

[12] Cortes JE, Kantarjian H, Shah NP (2012). Ponatinib in refractory Philadelphia chromosome-positive leukemias. N Engl J Med.

[13] Chan O, Talati C, Isenalumhe L (2020). Side-effects profile and outcomes of ponatinib in the treatment of chronic myeloid leukemia. Blood Adv.

[14] (2021). FDA: Highlights of Prescribing Information. https://www.accessdata.fda.gov/drugsatfda_docs/label/2020/203469s034lbl.pdf.

[15] Dorer DJ, Knickerbocker RK, Baccarani M, Cortes JE, Hochhaus A, Talpaz M, Haluska FG (2016). Impact of dose intensity of ponatinib on selected adverse events: multivariate analyses from a pooled population of clinical trial patients. Leuk Res.

